# Joint 2D–3D cross-pseudo supervision for carotid vessel wall segmentation

**DOI:** 10.3389/fcvm.2023.1203400

**Published:** 2023-11-17

**Authors:** Yahan Zhou, Lin Yang, Yuan Guo, Jing Xu, Yutong Li, Yongjiang Cai, Yuping Duan

**Affiliations:** ^1^ School of Mathematical Sciences, Beijing Normal University, Beijing, China; ^2^ School of Statistics and Mathematics, Zhejiang Gongshang University, Hangzhou, China; ^3^ Health Management Center, Peking University Shenzhen Hospital, Peking University, Shenzhen, China; ^4^ Center for Applied Mathematics, Tianjin University, Tianjin, China

**Keywords:** carotid artery wall, atherosclerosis, black-blood vessel wall MRI, semi-supervised learning, continuous prior, Graphical User Interface

## Abstract

**Introduction:**

The segmentation of the carotid vessel wall using black-blood magnetic resonance images was a crucial step in the diagnosis of atherosclerosis. The objective was to accurately isolate the region between the artery lumen and outer wall. Although supervised learning methods achieved remarkable accuracy in vessel segmentation, their effectiveness remained limited due to their reliance on extensive labeled data and human intervention. Furthermore, when confronted with three-dimensional datasets featuring insufficient and discontinuous label data, these learning-based approaches could lose their efficacy. In this paper, we proposed a novel Joint 2D–3D Cross-Pseudo Supervision (JCPS) method for accurate carotid vessel wall segmentation.

**Methods:**

In this study, a vascular center-of-gravity positioning module was developed to automatically estimate the region of blood vessels. To achieve accurate segmentation, we proposed a joint 2D–3D semi-supervised network to model the three-dimensional continuity of vascular structure. In addition, a novel loss function tailored for vessel segmentation was introduced, consisting of four components: supervision loss, cross-pseudo supervision loss, pseudo label supervision loss, and continuous supervision loss, all aimed at ensuring the accuracy and continuity of the vessel structure. In what followed, we also built up a user-friendly Graphical User Interface based on our JCPS method for end-users.

**Results:**

Our proposed JCPS method was evaluated using the Carotid Artery Vessel Wall Segmentation Challenge dataset to assess its performance. The experimental results clearly indicated that our approach surpassed the top 10 methods on the leaderboard, resulting in a significant enhancement in segmentation accuracy. Specifically, we achieved an average Dice similarity coefficient increase from 0.775 to 0.806 and an average quantitative score improvement from 0.837 to 0.850, demonstrating the effectiveness of our proposed JCPS method for carotid artery vessel wall segmentation.

**Conclusion:**

The experimental results suggested that the JCPS method had a high level of generalization performance by producing pseudo labels that were comparable with software annotations for data-imbalanced segmentation tasks.

## 1. Introduction

Cardio-cerebrovascular disease (CCVD) manifests as systemic vasculopathy affecting the heart and brain, making it a global public health concern and a leading cause of mortality. Vascular medical images are extensively used to visualize the three-dimensional (3D) morphology of cardiac and cerebral vessels, playing an essential role in the diagnosis and treatment of CCVD. Blood vessel segmentation is aimed at extracting well-defined vessel structures from these medical images. Therefore, computer-based automatic detection and segmentation of blood vessel walls are of great clinical significance, as they represent a crucial step in ensuring precise diagnosis, early intervention, and surgical planning for CCVD.

However, medical image segmentation has not been adequately handled due to the complexity and diversity of the medical images. Consequently, researchers have dedicated significant efforts to develop effective segmentation methods, including both traditional and deep learning–based approaches in recent years. Traditional image segmentation techniques, such as thresholding (1, 2), region growing method (3–5), active contour model (6, 7), and level set method (8–10), have been widely recognized. However, these methods have their limitations. They are often semi-automatic and rely on human input, making them prone to noise interference and intensity unevenness. Deep learning methods have shown remarkable performance in medical image segmentation tasks. For instance, the fully convolutional network (FCN) can take inputs of arbitrary sizes and produce correspondingly sized output with efficient inference and learning for image segmentation tasks. Since then, the FCN has been extensively used in the fields of medical image segmentation (11–13), e.g., the segmentation of breast tumors on MR images (13) and the segmentation of human torsos on CT images (12). However, the FCN suffered from issues such as inaccurate edges and loss of details. The U-Net architecture (14) used the jump connections to effectively realize the integration of features and performed more efficiently in training. Since then, it was widely used for medical image segmentation (15–18). To deal with small organs or tissues, a coarse-to-fine segmentation framework was established to enhance the accuracy by extracting regions of interest (ROI) during the coarse segmentation stage and using ROI as inputs for the fine segmentation network. These kinds of approaches have achieved satisfactory performance in various image segmentation tasks (19, 20) and were also successfully applied to handle vascular segmentation problems (21–24).

Indeed, the vessel segmentation had unique characteristics such as the significant imbalance of blood vessel proportions, complex structures of blood vessels, and difficulties in acquiring blood vessel labels. Samber et al. (25) applied a convolutional neural network (CNN) to segment the carotid artery after extensive manual preprocessing to improve carotid artery segmentation accuracy. Oliveira et al. (26) combined the multiscale analysis provided by the stationary wavelet transform with a multiscale FCN for the purpose of automatic vessel segmentation. Ni et al. (27) proposed a global channel attention network (GCA-Net) to segment intracranial blood vessels. Liu et al. (28) developed a novel residual depth-wise over-parameterized convolutional (ResDO-conv) network for automatic and accurate retinal vessel segmentation. Imran et al. (29) designed an intelligence-based automated shallow network with high performance and low cost named Feature Preserving Mesh Network (FPM-Net) for the accurate segmentation of retinal vessels. Tan et al. (30) proposed the U-Net using local phase congruency and orientation scores (UN-LPCOS), which showed a remarkable ability to identify and segment small retinal vessels. However, the aforementioned methods were all built up for dealing with 2D vessel segmentation tasks. Zhou et al. (31) proposed an approach that combined a voxel-based fully convolution network (Voxel-FCN) and a continuous max-flow module to automatically segment the carotid vessel wall. Tetteh et al. (32) presented the DeepVesselNet to extract vessel trees in 3D angiographic volumes. Xia et al. (33) proposed an edge-reinforced network (ER-Net) for 3D vessel-like structure segmentation, which incorporates a reverse edge attention module. Alblas et al. (34) formulated the vessel wall segmentation as a multi-task regression problem in polar coordinates to automatically segment the carotid artery wall with high accuracy. However, the performance of these methods was hindered when insufficient labeled data were available. As such, semi-supervised segmentation methods became increasingly popular to alleviate the demand for labeled data, which could be broadly classified into entropy-minimization–based methods (35) and consistency determination–based methods (36–39). Recently, a novel approach known as cross-pseudo supervision (CPS) has emerged to enhance performance in semi-supervised learning problems (40, 41). The CPS method enforces consistency among slightly different network outputs, leading to satisfactory results even with limited labeled data. More importantly, the CPS method effectively avoids confronting the strong coupling between the teacher and student networks (42).

In this paper, we presented a novel coarse-to-fine vessel wall segmentation method. In the coarse segmentation stage, we developed a modified Deeplabv3+ network to estimate both the vessel location and signed distance function. Based on the coarse segmentation, we calculated the location of the blood vessel’s center of gravity using the first-order moment method. This information was then utilized to crop the original images, specifically selecting the ROI that contained the vessels. In the fine segmentation stage, we proposed a joint 2D–3D CPS network to ideally exploit the spatial information of 3D volumes and used the continuity prior of blood vessels, which helped enhance the blood vessel features and improved the segmentation accuracy. It is worth mentioning that the CPS operation involved both labeled and unlabeled data, which improved the generalizability using the lower cost of manual annotation. In comparison to existing coarse-to-fine methods, our model incorporated both the position of the center of gravity and the continuity of the target blood vessel to enhance the utilization of carotid artery features. The proposed method was evaluated on the 3D carotid black-blood MRI dataset obtained from the Carotid Artery Vessel Wall Segmentation Challenge, which was a typical semi-supervised segmentation task with only around 20% labeled data. Through numerical experiments, we were able to demonstrate that our JCPS method surpassed the state-of-the-art results on the competition’s leader board, exhibiting a significant improvement in segmentation accuracy when compared to both the baseline U-Net model and single CPS model. Furthermore, we designed an effective and user-friendly Graphical User Interface (GUI) for the automated segmentation of MRI images of black-blood carotid arteries, aimed at providing valuable assistance to clinicians in their diagnostic.

The rest of this paper is organized as follows. Section 2. introduces our joint 2D–3D cross-pseudo supervision method, including coarse and fine segmentation models, a loss function, and implementation details. Section 3. presents experimental results and ablation studies. We briefly discuss the proposed approach and conclude with a summary and possible future work in Section 4.

## 2. Materials and methods

### 2.1. Data source

The training set and test set data used in this study were both from the Carotid Artery Vessel Wall Segmentation Challenge, in which 25 cases with various carotid vessel wall conditions were used as the training set, and the other 25 cases with various carotid vessel wall conditions were used as the test set. A total of 12,920 vessel wall images of sufficient quality in the training set (2,584 images with manual contour labels) were used for training, and a total of 2,412 images with manual contour labels in the test set were used for testing. Each vessel wall image is an axial slice of a carotid black-blood MRI image, and the size of each original image is of 720×720 in order to facilitate subsequent evaluation and meet the Carotid Artery Vessel Wall Segmentation Challenge.

### 2.2. Our approach

**Figure 1:**
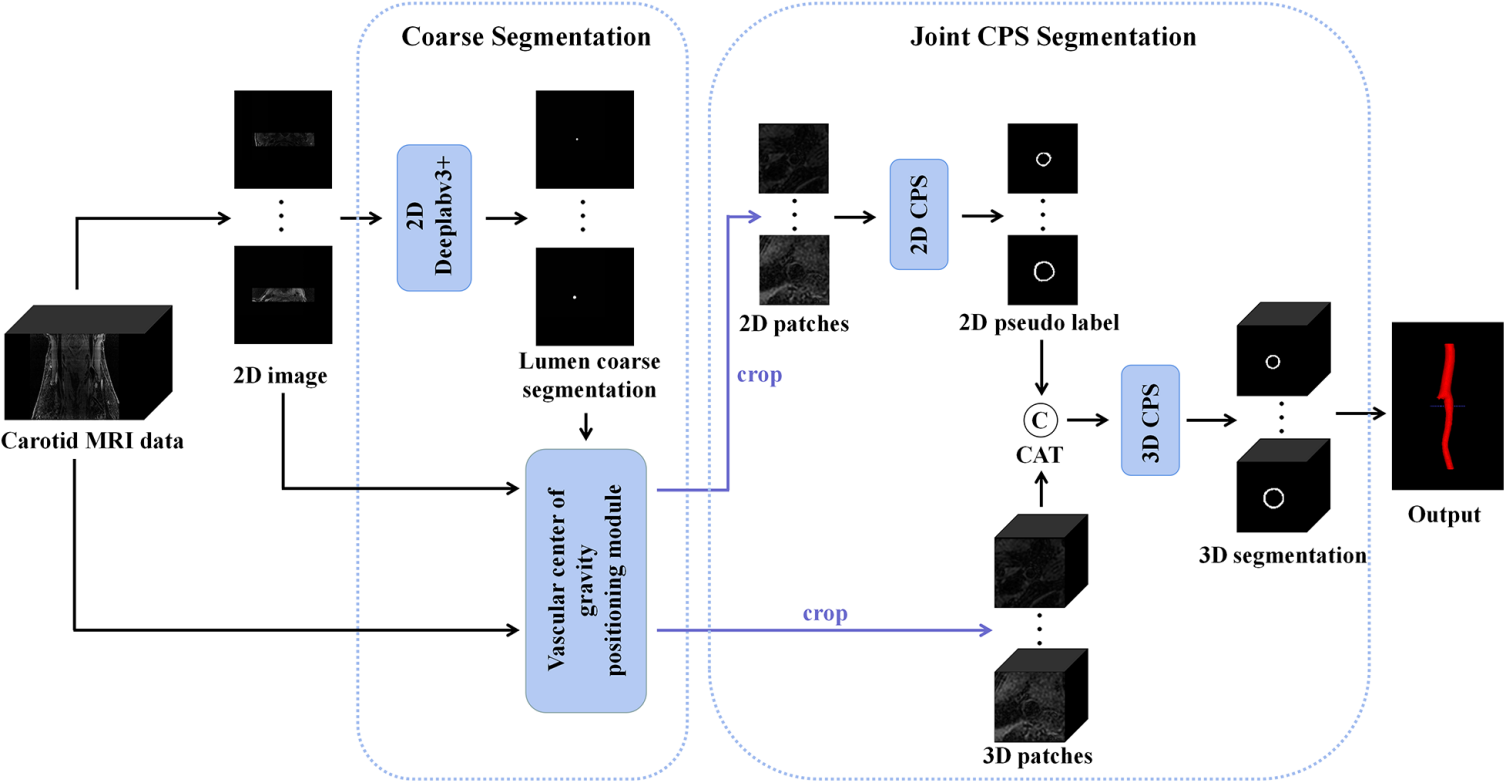
An overview of the proposed JCPS framework, where CAT is short for concatenation. In the coarse segmentation model, a 2D Deeplabv3+ network was employed to locate objects in high-resolution images. The vascular center-of-gravity positioning module, derived from the lumen coarse segmentation, was utilized to identify the vascular center of gravity in both 2D and 3D original images. For the fine segmentation model, the CPS network was adopted, enabling efficient utilization of limited labeled data and a large amount of unlabeled data to achieve precise segmentation.

The proposed automatic carotid artery vessel wall segmentation approach, known as the Joint 2D–3D Cross-Pseudo Supervision (JCPS), comprised two stages, as illustrated in Figure 1. The coarse segmentation model consisted of a vascular center-of-gravity positioning model, and the fine segmentation model consisted of a joint 2D–3D CPS network.

#### 2.2.1. Coarse segmentation

Since the target vessel occupied only a small fraction of the whole image and varied in sizes and locations in 2D axial slices, we needed to automatically determine the approximated location of the center of gravity for the blood vessel. This was crucial for providing a region of interest specific to the local vessel area, which would be utilized for subsequent vessel wall segmentation. To achieve this, we developed a vascular center-of-gravity positioning module within the coarse segmentation model to estimate the center of the vessel. The backbone of the coarse segmentation model was chosen as DeepLabv3+ (43), which had been commonly used for medical segmentation (44, 45).

**Figure 2:**
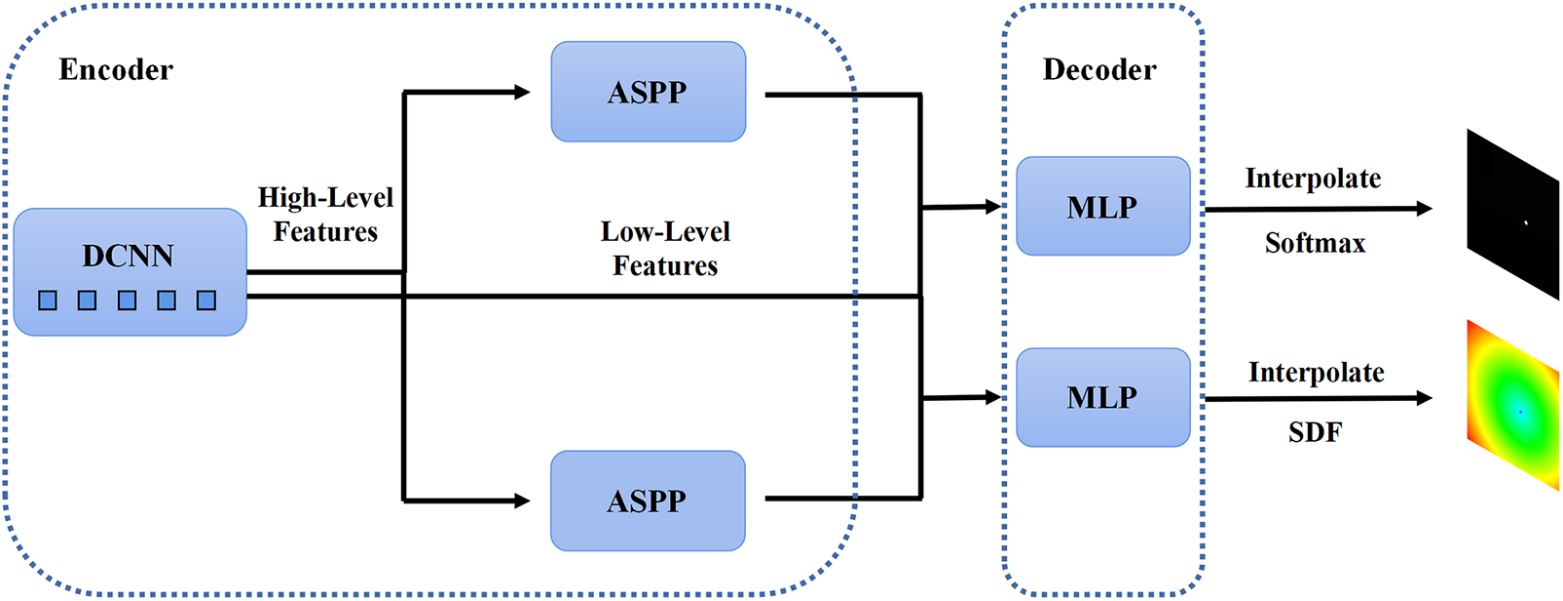
The structure of the 2D Deeplabv3+ network with the encoder module and the decoder module. The high-level feature information is first transferred through the deep convolutional network into two parallel feature pyramid modules, and each enters the Multi-Layer Perceptron (MLP) of the decoder module, which simultaneously outputs the pixel-level classification and the corresponding signed distance function.

**Figure 3:**
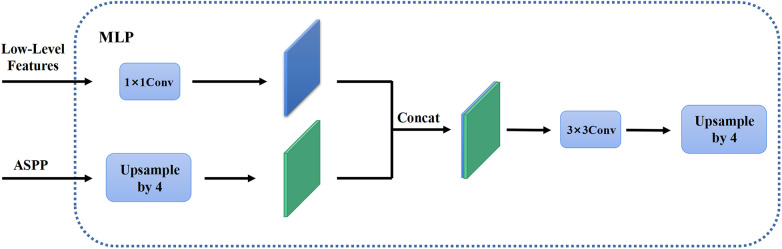
The MLP module in the decoder. The high-level feature information through the feature pyramid module is upsampled by quadruple interpolation and stacked with the low-level feature information in the channel dimension, while the output is the same as the original input image resolution after a 3×3 convolution and a quadruple upsampling.

In the first stage, we identified 2D slices with the sufficient image quality from 3D carotid black-blood MRI images $I_{3D}\in R^{D\times H\times W}$, where D, H, and W represent the depth, height, and width of the 3D volume, respectively. The input and output of the coarse segmentation model were represented as $I_{2D}\in R^{H\times W}$ and $Q_{2D}\in R^{H\times W}$, respectively. Different from the classical Deeplabv3+ network, our approach involved learning both the pixel-level classification task and the signed distance function. These components were utilized to achieve binary classification results for the lumen area and to accurately capture the lumen boundary, respectively. For a detailed overview of the network architecture, please refer to Figure 2. The input image was processed utilizing a deep convolutional neural network (DCNN) to extract both low-level and high-level features. Following that, the high-level features were fed into the Atrous Spatial Pyramid Pooling (ASPP) module, which consists of parallel dilated convolutional layers and pooling layers to extend the receptive field. It is capable of extracting relevant features from original images with a relatively low proportion of vessel regions, and subsequently merging them at different scales, thereby enhancing the accuracy of the coarse segmentation stage. Within the decoder, the Multi-Layer Perceptron (MLP) module concatenated the low-level and high-level features derived from the encoder. Subsequently, the outputs were restored to the original image resolution by employing interpolation and upsampling techniques. For a detailed illustration of the network structure, please refer to Figure 3. Based on the coarse segmentation, we used the vessel center-of-gravity positioning model to crop the data into 2D or 3D patches, which were utilized as inputs for the fine segmentation model. Subsequently, the fine segmentation model accurately predicted binary labels for 3D carotid black-blood MRI volumes, with “0” representing the background and “1” denoting the vessel wall.

#### 2.2.2. 2D CPS network

To calculate the center of gravity of the 2D lumen area, we utilized the first-order moment as follows

(1) $$G_{2D}=g_{2D}(Q_{2D}),$$

with

$$g_{2D}(Q_{2D})=\left(\frac{\sum_{I}\sum_{J}i\cdot Q_{2D}(i,j)}{\sum_{I}\sum_{J}Q_{2D}(i,j)},\frac{\sum_{I}\sum_{J}j\cdot Q_{2D}(i,j)}{\sum_{I}\sum_{J}Q_{2D}(i,j)}\right),$$

where $Q_{2D}(i,j)$ represents the gray value of the binary segmentation map $Q_{2D}$ at point (i,j). Obviously, the center of gravity of the segmented lumen was an approximation for the centerline of the vessels. Subsequently, the estimated center of gravity was used to crop local patches $X_{2D}\in R^{h\times w}$ with a fixed size h×w. These patches were then employed as inputs for the fine segmentation model.

**Figure 4:**
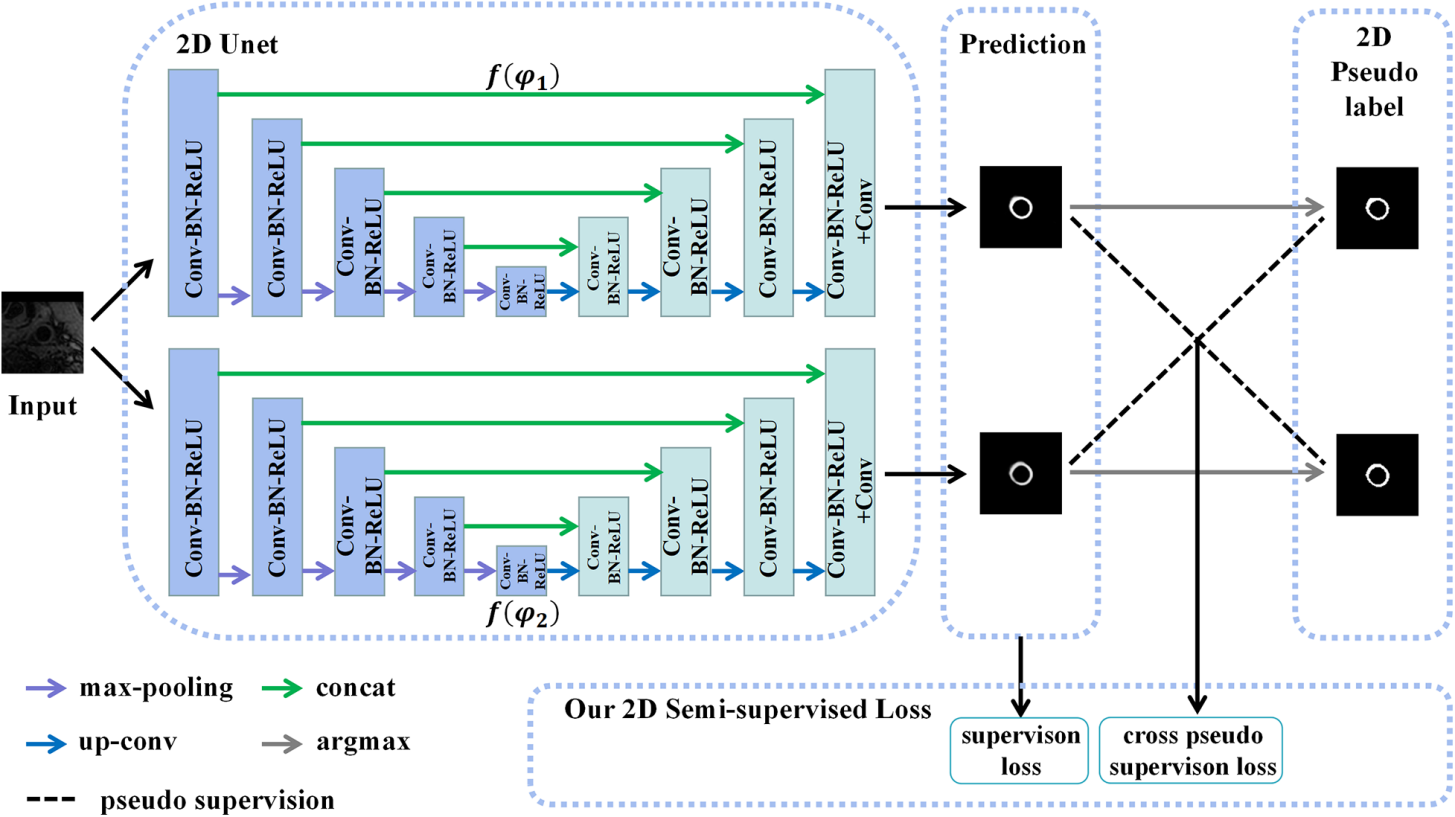
The structure of the 2D CPS network, which consists of two 2D U-Net networks with varying parameters. Our loss function comprises the supervision loss and cross-pseudo supervision loss, where supervision loss represents the loss between the output of U-Net and the ground truth, and cross-pseudo supervision loss represents the loss of mutual supervision of the pseudo labels of the two U-Net outputs.

Assuming that the manual labels were randomly distributed in the 3D carotid black-blood MRI volumes, comprising approximately 20% of the total slices, we endeavored to exploit a limited amount of 2D labeled data and a substantial amount of 2D unlabeled data to generate more precise pseudo labels for the latter. To achieve this, we employed a 2D semi-supervised method CPS to integrate the pseudo labels and consistency regularization, thereby maximizing the utilization of both labeled and unlabeled data. Specifically, the U-Net architecture was adopted as the backbone of the CPS network, as depicted in Figure 4. The U-Net consisted of a contracting path and an expansive path. Notably, the number of channels in the network was halved compared to the traditional U-Net. Each convolutional layer (Conv) was followed by a batch normalization (BN) and a rectification linear unit (ReLU), denoted as a composite layer (Conv-BN-ReLU).

As depicted in Figure 4, two U-Net networks, denoted as $f(\varphi_{1})$ and $f(\varphi_{2})$, were initially generated. These networks shared the same structure but had different initialization parameter. The patches $X_{2D}$, obtained from the coarse segmentation stage and containing both labeled and unlabeled data, served as inputs for both U-Nets. Their objective was to estimate the segmentation confidence maps $P_{2D}^{n}\in R^{C\times h\times w}\;(n=1,2)$, which can be expressed as

(2) $$P_{2D}^{n}=f(X_{2D};\varphi_{n}),$$

where C represented the number of categories, i.e., the images were divided into C categories. The corresponding one-hot labels $S_{2D}^{n}\in R^{h\times w}\;(n=1,2)$ were then obtained through the argmax operation. These labels were considered the pseudo labels predicted by the two networks. During the training of unlabeled data, we adopted the method of pseudo-label mutual supervised learning, where the pseudo labels $S_{2D}^{1}$ were used to supervise $P_{2D}^{2}$, and the pseudo labels $S_{2D}^{2}$ were used to supervise $P_{2D}^{1}$. The goal was to enforce a high degree of consistency between the predictions of the two perturbed networks. Subsequently, the continuous 2D pseudo labels $S_{2D}\in R^{h\times w}$ obtained after sufficient training were concatenated as additional inputs for the 3D CPS network. These pseudo labels also provided auxiliary supervision for the outputs of the 3D CPS network.

#### 2.2.3. 3D CPS network

**Figure 5:**
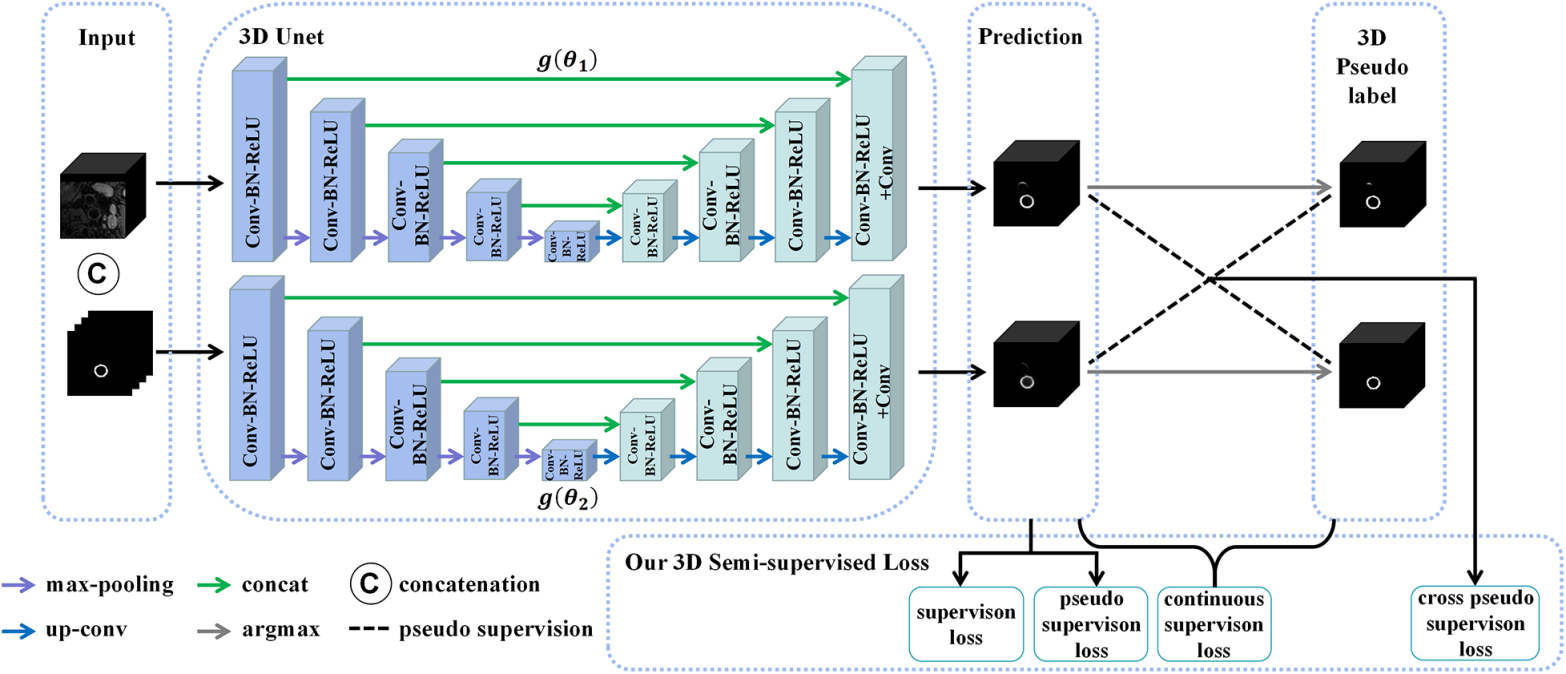
The structure of the 3D CPS network, where CAT is short for concatenation. The original image patches and the pseudo labels output by the 2D CPS network are concatenated to form the input of the 3D CPS net. It consists of two 3D U-Net networks with varying parameters. The loss function of the 3D CPS network consists of four parts: the supervision loss, pseudo supervision loss, continuous supervision loss, and cross-pseudo supervision loss.

Although the 2D CPS model estimated the 2D pseudo labels, it lacked the modeling of three-dimensional continuity. On the other hand, employing 3D methods that take 3D images as inputs often incurs high computational costs. To mitigate such issues, the use of smaller 3D patches can be considered to balance the performance and computational efficiency. Thus, we proposed a novel method for acquiring 3D patches by utilizing the vascular center-of-gravity positioning model and 2D pseudo labels to extract the relevant local vascular regions of interest. In addition, an overlapping sliding window approach was employed to preserve more contextual information within the extracted patches. Firstly, we split the 3D volumes into a series of small-size 3D patches $J_{3D}\in R^{d\times H\times W}$, where d represented the depth of the desired 3D patch. For each $J_{3D}$, there was a corresponding 3D lumen binary segmentation map $Q_{3D}\in R^{d\times H\times W}$, which was obtained by gathering the 2D lumen coarse segmentation $Q_{2D}$. The vascular center of gravity $G_{3D}$ of the 3D image $J_{3D}$ was calculated using $Q_{3D}$ according to the following equation:

(3) $$G_{3D}=g_{3D}(Q_{3D}),$$

where $g_{3D}$ denoted the 3D first-order moment function, which was a direct extension of the equation (1). Specifically, only the x-axis and y-axis coordinates of $G_{3D}$ needed to be determined since the patch depth had already been fixed to d. Therefore, we used the position information of the vascular center of gravity $G_{3D}$ to crop the input patch $J_{3D}$ into $X_{3D}\in R^{d\times h\times w}$, where the sizes were d×h×w. Finally, the obtained 3D patches and pseudo labels were used as inputs for the newly proposed 3D CPS network to estimate the segmentation results. The specific architecture of this network is illustrated in Figure 5.

Similar to the 2D CPS model, the 3D CPS network was constructed using two 3D U-Net networks, denoted as $g(\theta_{1})$ and $g(\theta_{2})$, which had identical structures but different parameters. The input of the 3D CPS network consisted of both the 3D patches and the pseudo labels estimated by the 2D CPS. The output of the 3D CPS network was represented by the confidence map $P_{3D}^{m}\in R^{C\times d\times h\times w}(m=1,2)$. Therefore, the relationship could be expressed as follows:

(4) $$P_{3D}^{m}=g(X_{3D},P_{2D};\theta_{m}).$$

Consequently, we obtained the corresponding pseudo labels $S_{3D}^{m}\in R^{d\times h\times w}\;(m=1,2)$ through the argmax operation. In contrast to the 2D CPS model, the limited availability of 2D labeled data within the 3D patches, which accounted for less than 10%, posed difficulty for the semi-supervised network CPS to achieve accurate segmentation. To address this challenge, we additionally used the pseudo labels obtained by the 2D CPS network to supervise the predictions of the 3D CPS network. Simultaneously, the pseudo-label supervised learning enforced the prediction of 3D CPS to be of high consistency with the 2D CPS model. In addition, we exploited the spatial continuity of the vessels in order to enhance the plausibility of the predictions made by the 3D CPS network.

#### 2.2.4. Loss function

In the following, we will discuss the loss functions used for coarse segmentation and fine segmentation, respectively.

In the coarse segmentation stage, the network output the classification and signed distance function (SDF) simultaneously. We used the Focal Tversky (FT) loss function (46) to calculate the loss of the pixel-wise classification, given as follows:

$$L_{\mathrm{FT}}=(1-L_{T}{)}^{\gamma },$$

with

$$L_{T}=\frac{\left|P\cap Y\right|}{\left|P\cap Y|+\alpha |P-Y|+\beta |Y-P\right|},$$

where $L_{T}$ represents the Tversky Loss, P and Y represent the predicted pixel-level classification results and ground truth, and α and β controlled the proportion of false positives and false negatives, respectively. As can be seen, the Focal Tversky loss introduced a focal mechanism based on the Tversky index. Compared to the traditional cross-entropy loss function, it was proven to be better suited for addressing class imbalance issues in image segmentation. In addition, it can enhance penalty on boundary regions and suppress the classification of pixels being misclassified. Therefore, we adopted the Focal Tversky loss to address the challenging vessel segmentation problem in coarse segmentation.

The SDF reflected the position information and boundary information of the segmented lumen, which was defined as follows:

$$\varphi (x)=\left\{ \begin{array}{cc} -\inf_{y\in \partial V}\|x-y{\|}_{2},\; & \text{if }\;x\in V;\;\\ 0,\; & \text{if }\;x\in \partial V;\;\\ \inf_{y\in \partial V}\|x-y{\|}_{2},\; & \text{if }\;x\in \Omega \setminus V;\; \end{array}\right.$$

where V represents the vascular area, y was the point on the border of the vascular area, $\varphi \;:\;\Omega \subset R^{2}\to R$, the signed distance function was expressed as the infimum of the minimum value to the border of the vascular area for a given point x. Thus, the loss function for the coarse segmentation stage consisted of the following two terms:

$$L=L_{\mathrm{FT}}+L_{\mathrm{SDF}}.$$

In order to balance the loss contributions from both tasks, we used the homoscedastic uncertainty for weighting a dual-task loss function as follows:

(5) $$L(\sigma_{1},\sigma_{2})=\frac{1}{\sigma_{1}^{2}}L_{\mathrm{FT}}+\frac{1}{\sigma_{2}^{2}}L_{\mathrm{SDF}}+\log\sigma_{1}\sigma_{2},$$

where parameters $\sigma_{1}$ and $\sigma_{2}$ corresponded to the homoscedastic uncertainties of the Focal Tversky loss and the signed distance function loss, regarding the classification task and the regression task, respectively. By minimizing the loss L and the noise variables $\sigma_{1}$, $\sigma_{2}$, task-specific losses could be balanced during the training process.

The training of 2D CPS consisted of the supervision loss $L_{2D}^{s}$ and cross-pseudo supervision loss $L_{2D}^{\mathrm{cps}}$ such as

(6) $$L_{2D}=L_{2D}^{s}+\lambda_{0}L_{2D}^{\mathrm{cps}},$$

where $\lambda_{0}$ was the trade-off weight. The supervision loss for the labeled data included the cross-entropy and dice loss as given below:

$$L_{2D}^{s}=\frac{1}{2}\sum_{n=1}^{2}(l_{\mathrm{ce}}(Y^{n},P_{2D}^{n})+l_{d}(Y^{n},P_{2D}^{n})),$$

where $Y^{n}$ represented the ground truth, $l_{\mathrm{ce}}$ was the cross-entropy loss, and $l_{d}$ was the dice loss. In addition, the cross-pseudo supervision loss formula for labeled data and unlabeled data was also considered

$$L_{2D}^{\mathrm{cps}}=l_{\mathrm{ce}}(S_{2D}^{2},P_{2D}^{1})+l_{\mathrm{ce}}(S_{2D}^{1},P_{2D}^{2}).$$

In addition, the loss function for 3D CPS included the supervision loss $L_{3D}^{s}$, the cross-pseudo supervision loss $L_{3D}^{\mathrm{cps}}$, the pseudo label supervision loss $L_{3D}^{\mathrm{ps}}$, and the continuous supervision loss $L_{3D}^{\mathrm{cs}}$, which was defined as follows:

(7) $$L_{3D}=L_{3D}^{s}+\lambda_{1}L_{3D}^{\mathrm{cps}}+\lambda_{2}L_{3D}^{\mathrm{ps}}+\lambda_{3}L_{3D}^{\mathrm{cs}},$$

where $\lambda_{1}$ and $\lambda_{3}$ were the trade-off weights, and $\lambda_{2}$ was the pseudo label weight. Because the labels were in 2D format, the supervision loss construction for the labels for the 3D CPS network was the same as for the 2D CPS network, i.e.,

$$L_{3D}^{s}=\frac{1}{2|A|}\sum_{i\in A}\sum_{m=1}^{2}(l_{\mathrm{ce}}(Y_{i}^{m},P_{3D}^{m}(i))+l_{d}(Y_{i}^{m},P_{3D}^{m}(i))),$$

where A was the set of labels, $Y_{i}^{m}$ represented the ground truth, and $P_{3D}^{m}(i)$ represents the ith layer of the output $P_{3D}^{m}$ of the 3D CPS network. In addition, the cross-pseudo supervision loss was defined as follows

$$L_{3D}^{\mathrm{cps}}=l_{\mathrm{ce}}(S_{3D}^{2},P_{3D}^{1})+l_{\mathrm{ce}}(S_{3D}^{1},P_{3D}^{2}),$$

where $S_{3D}^{m}$ represents the pseudo label estimated by the 3D CPS network for m=1,2. The pseudo-label supervised loss formula for unlabeled data was described as

$$L_{3D}^{\mathrm{ps}}=\frac{1}{2|B|}\sum_{i\in B}\sum_{m=1}^{2}(l_{\mathrm{ce}}(S_{2D},P_{3D}^{m}(i))+l_{d}(S_{2D},P_{3D}^{m}(i))),$$

where B was the unlabeled dataset, and $S_{2D}$ was the pseudo labels of the segmentation from the 2D CPS network. Finally, the continuous supervision loss was defined as follows:

$$L_{3D}^{\mathrm{cs}}=\frac{1}{2}\sum_{m=1}^{2}\left(\sum_{i=1}^{d-1}l_{\mathrm{ce}}(S_{3D}^{m}(i+1),P_{3D}^{m}(i))+\sum_{i=2}^{d}l_{\mathrm{ce}}(S_{3D}^{m}(i-1),P_{3D}^{m}(i))\right).$$

### 2.3. Evaluation metrics

In the testing phase, the performance of the proposed method was evaluated using manually corrected ground truth. The segmentation effectiveness of the vessel wall, lumen, and outer wall was assessed using the following designed quantitative metrics (QS), the Dice Similarity Coefficient (DSC) of the lumen region (${\mathrm{DSC}}^{L}$), and the DSC of the wall region (${\mathrm{DSC}}^{W}$). QS was calculated based on six additional indicators: the DSC of the vessel wall region, Lumen area difference (Lad), Wall area difference (Wad), Normalized wall index difference (Nwid), Hausdorff distance on lumen normalized by radius (Hdol), and Hausdorff distance on wall normalized by radius (Hdow). The calculation of QS was as follows:

QS=0.5×DSC+0.1×(f(Lad)+f(Wad))+0.2×f(Nwid)+0.05×(f(Hdol)+f(Hdow)),

where f(x)=max(0,1−x). As an ensemble similarity measure, DSC was computed to assess the similarity between the vessel wall segmentation result and the ground truth, which was defined as follows:

$$\mathrm{DSC}=\frac{2(X\cap Y)}{X+Y}.$$

where X and Y represent the binary vessel wall segmentation result and ground truth, respectively. Therefore, DSC equaled 1 when the segmentation result was the same as the ground truth. The Lad and Wad calculated the area difference between the lumen and outer wall and the ground truth, respectively, which were defined as follows:

$$\mathrm{Lad}=\frac{\left|{XA}^{L}-{YA}^{L}\right|}{{YA}^{L}},\;\mathrm{Wad}=\frac{\left|{XA}^{W}-{YA}^{W}\right|}{{YA}^{W}}.$$

where ${XA}^{L}$, ${XA}^{W}$, ${YA}^{L}$, and ${YA}^{W}$ represent the area of the lumen segmentation, the area of the outer wall segmentation, and their corresponding ground truth areas, respectively. In addition, the Nwid represented the difference between the normalized outer wall area and the normalized outer wall ground truth area using the following formula:

$$\mathrm{Nwid}=\frac{|\frac{{XA}^{W}-{XA}^{L}}{{XA}^{L}}-\frac{{YA}^{W}-{YA}^{L}}{{YA}^{L}}|}{\frac{{YA}^{W}-{YA}^{L}}{{YA}^{L}}}.$$

The Hdol and Hdow were calculated by the Hausdorff distance between the contours of the lumen and outer wall to the ground truth, respectively, such as

$$\mathrm{Hdol}=\frac{\max(h({XO}^{L},{YO}^{L}),h({YO}^{L},{XO}^{L}))}{\sqrt{{XA}^{L}/\pi }},$$

and

$$\mathrm{Hdow}=\frac{\max(h({XO}^{W},{YO}^{W}),h({YO}^{W},{XO}^{W}))}{\sqrt{{XA}^{W}/\pi }},$$

where $h(B,C)=\max_{b\in B}\{\min_{c\in C}\|b-c\|\}$, and ${XO}^{L}$, ${XO}^{W}$, ${YO}^{L}$, and ${YO}^{W}$ represent the contour point set of the lumen segmentation result, the contour point set of the outer wall segmentation result, and their corresponding ground truth contour point sets, respectively.

## 3. Experiments and results

Our method was implemented by using PyTorch, and all experiments were performed on a server with one NVIDIA Geforce RTX 3090 Founders Edition GPU. In the coarse segmentation stage, the total training time was 12 h. In the fine segmentation stage, the total training time was 7 h.

### 3.1. Data processing

**Figure 6:**
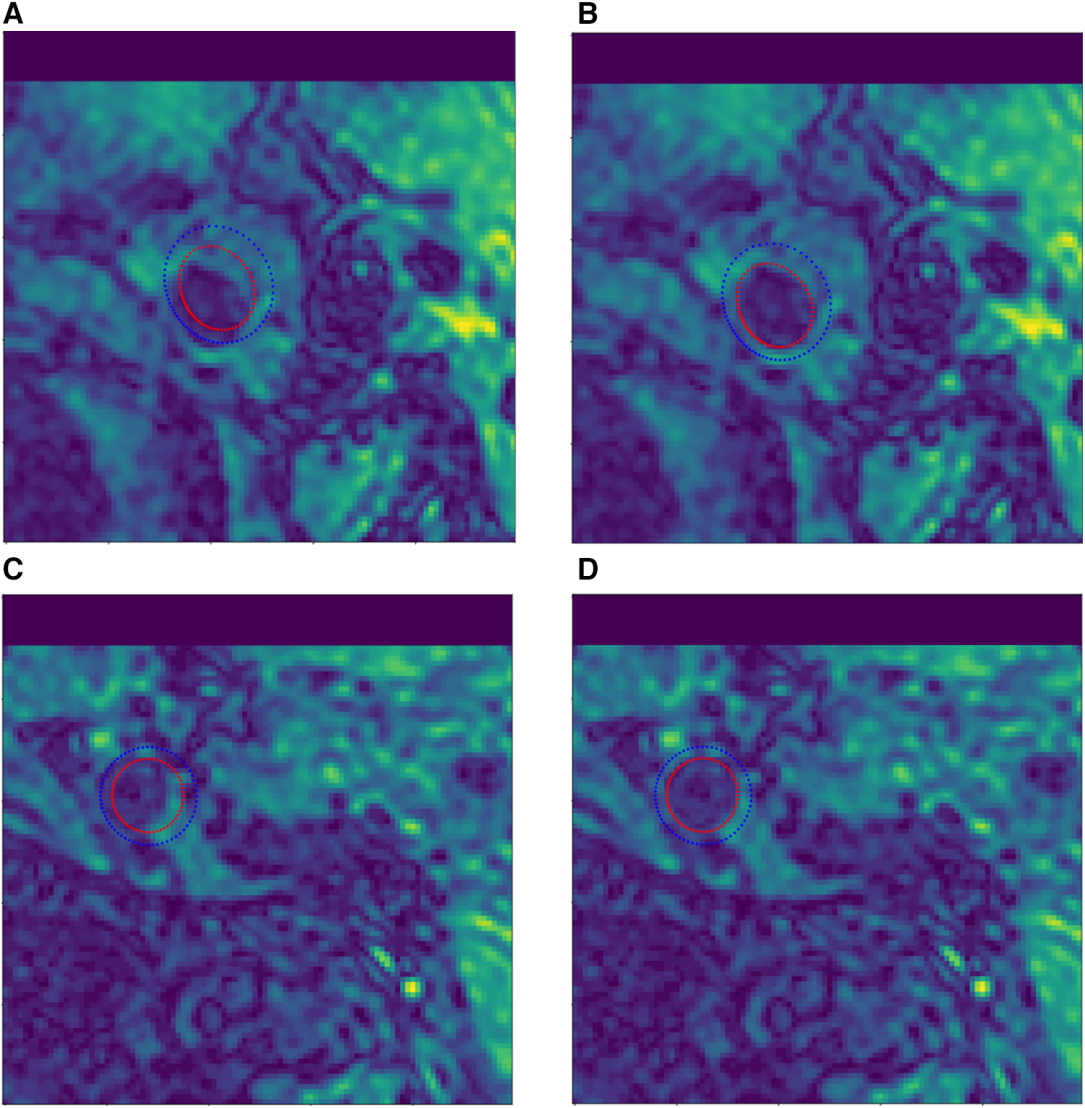
Example of manual contour label correction. (**A**,**C**) Test set label data with offset error. (**B**,**D**) Manually corrected test set label data.

Manual vessel contour labels were given by a customized vessel wall annotation software (CASCADE), so that some labels in the test set had a certain offset error, as shown in Figure 6. To address this issue, we manually corrected the label images to eliminate the offset errors. Specifically, a total of 526 labels with offset errors were manually adjusted to achieve the closest approximation to the ground truth, as shown in Figure 6(B,D).

### 3.2. Implementation details

The training details of our proposed JCPS network are described as follows. In the coarse segmentation stage, a Deeplabv3+ coarse segmentation network was trained, and its input patch size was the original resolution H×W, where H and W were both set to 720. The epoch number and batch size were set to 400 and 12, respectively. The Deeplabv3+ was optimized using an Adam optimizer, with a learning rate of 0.001, multiplied by 0.9 in iterations of 1,000. In the fine segmentation stage, a joint 2D–3D CPS network was trained to finely segment the vessel wall. For the 2D CPS network in JCPS, the input patch size was h×w, where h and w were both set to 96. The iteration number, batch size, and batch size of the labeled data were set to 30,000, 4, and 2, respectively. We employed the Poly learning rate strategy, where the learning rate was set to 0.01 and was changed by the initial learning rate multiplied by $(1-\text{iter}/\text{max}\_\text{iter}{)}^{0.9}$ for each iteration. In addition, we employed mini-batch stochastic gradient descent (SGD) with momentum to train 2D CPS, where the momentum was fixed at 0.9 and weight decay was set to 0.0001. For the 3D CPS network in JCPS, the input patch size was d×h×w, where d, h, and w were set to 32, 96, and 96, respectively. The iteration number, batch size, and batch size of the labeled data were set to 30,000, 4, and 2, respectively. The settings of the learning rate strategy and SGD were the same as in 2D CPS. In the loss function of the coarse segmentation stage, we set the weights as α=0.7, β=0.3, and γ=0.7. In the loss function of the fine segmentation stage, we empirically set the weights as $\lambda_{0}=\lambda_{1}=\lambda_{3}=e^{-5(1-t{)}^{2}},t=\text{epoch}/\text{max}\_\text{epoch}\in [0,1]$, which were a weight ramp-up equation (37) that increased with time, and $\lambda_{2}=1$. In particular, the parameter settings of all variants of our method were the same as those described above.

Note that the erroneous segmentation in the coarse segmentation may affect the selection of central points and subsequently impact the fine segmentation stage. The failure in the first stage can be roughly divided into three cases: (1) there are scattered fragments around the vessel wall, causing the center point to deviate from its geometric center; (2) due to the inability of coarse segmentation to accurately distinguish between internal and external carotid arteries at the bifurcation of blood vessels, the central point is located in the external carotid artery region; (3) in areas of carotid artery stenosis, especially extremely narrow areas, the coarse segmentation may not even be able to identify vascular, thus unable to locate the center point. Therefore, we applied the morphological post-processing to the results of the coarse segmentation. We eliminated fragmented regions in the coarse segmentation results by selecting the largest connected region. In the bifurcation area of the carotid artery, we used the position of the center point before and after the bifurcation to estimate the correct center point relying on the spatial continuity of vessels. Finally, we used the segmentation results of regular regions to interpolate the narrow regions.

### 3.3. Performance on the test dataset

**Figure 7:**
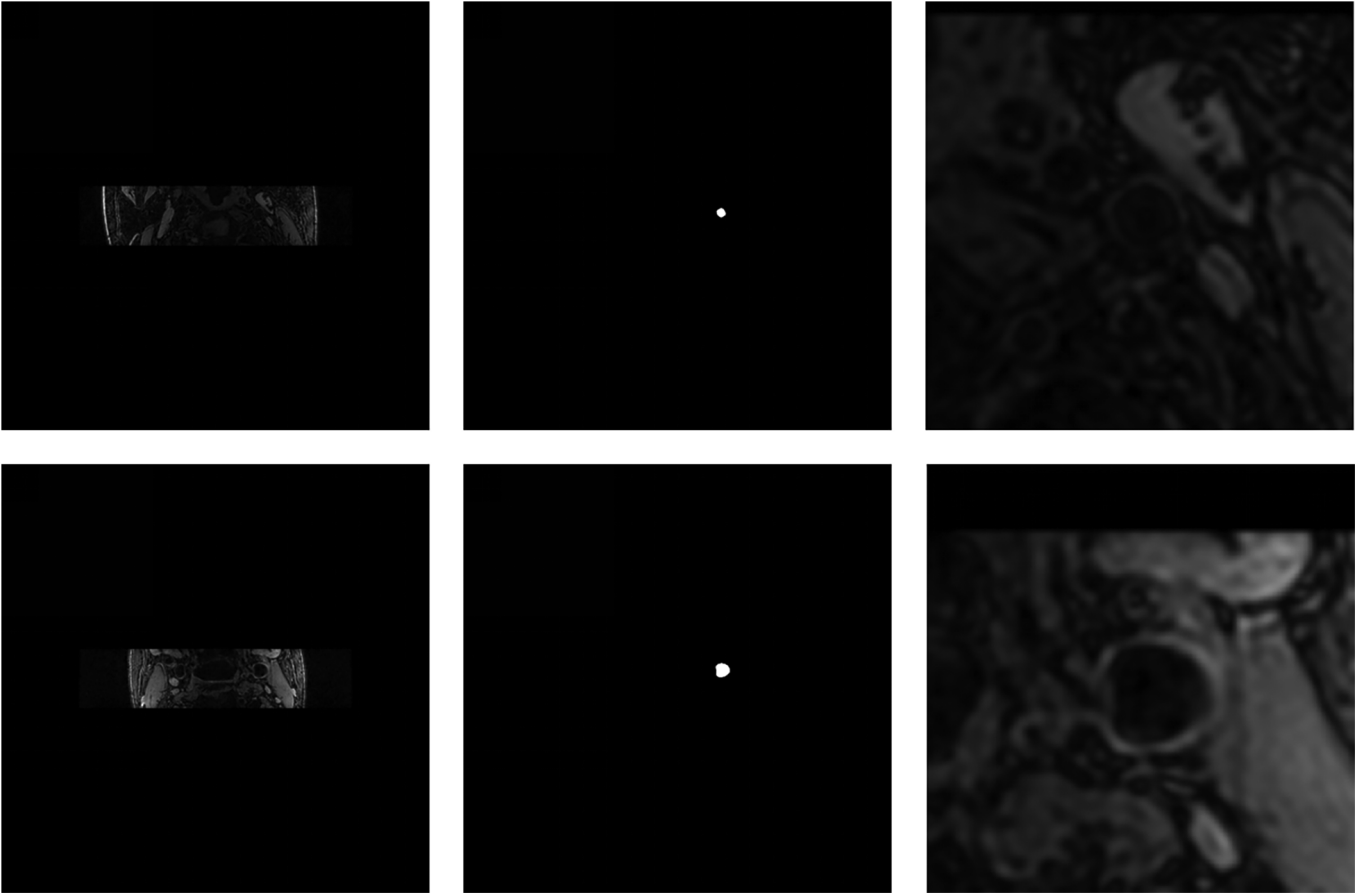
Visualization of the coarse segmentation. The first column are the original-resolution MRI images, the middle column are the centers of gravity estimated by coarse segmentation, and the last column are local patches obtained after passing through the vascular center-of-gravity positioning model.

In the first place, we used coarse segmentation to estimate the center of gravity and the local patches. As shown in Figure 7, our modified Deeplabv3+ model accurately identified the center of gravity in all slices. According to statistical analysis, we found that the diameter of carotid artery vessels is smaller than 64 pixels. Therefore, we set the patch size to 96×96 to capture sufficient information on the carotid vessels. Furthermore, we also validated that the segmentation results using 96×96 sized patches were optimal in numerical experiments.

In the fine segmentation stage, we evaluated the segmentation performance of our proposed method using the public 3D carotid black-blood MRI dataset. The segmentation accuracy of the top four methods on the leaderboard, as well as our method, is presented in Table 1. The results clearly demonstrated that our method surpassed the top-ranked team by more than 1% on quantitative scoring metrics and 3% on the Dice coefficient, while also surpassing other teams by a significant margin. In addition, the Lad, Wad, and Nwid indicators indicated a substantial reduction in errors within the segmented area using our JCPS model. Although the Hdol and Hdow indicators were slightly higher than those of the top-ranked team, the overall performance of our JCPS model was superior to all others.

**Table 1:** Performance of carotid vessel wall segmentation in comparison to the other top four teams.

	DSC	Lad	Wad	Nwid	Hdol	Hdow	QS
Team 1	0.775	0.086	0.072	0.080	**0.246**	**0.215**	0.837
Team 2	0.761	0.064	0.075	0.079	0.554	0.515	0.728
Team 3	0.736	0.089	0.136	0.139	0.366	0.358	0.727
Team 4	0.697	0.170	0.144	0.130	0.407	0.361	0.694
Ours	**0.806**	**0.063**	**0.068**	**0.054**	0.305	0.297	**0.850**

Teams 1–4 are the top four methods in the Carotid Artery Vessel Wall Segmentation Challenge. Evaluation indicators include DSC of the vessel wall region, Lad, Wad, Nwid, Hdol, Hdow, and QS.

The bold values represent the optimal results achieved in the respective columns for the indicators.

**Figure 8:**
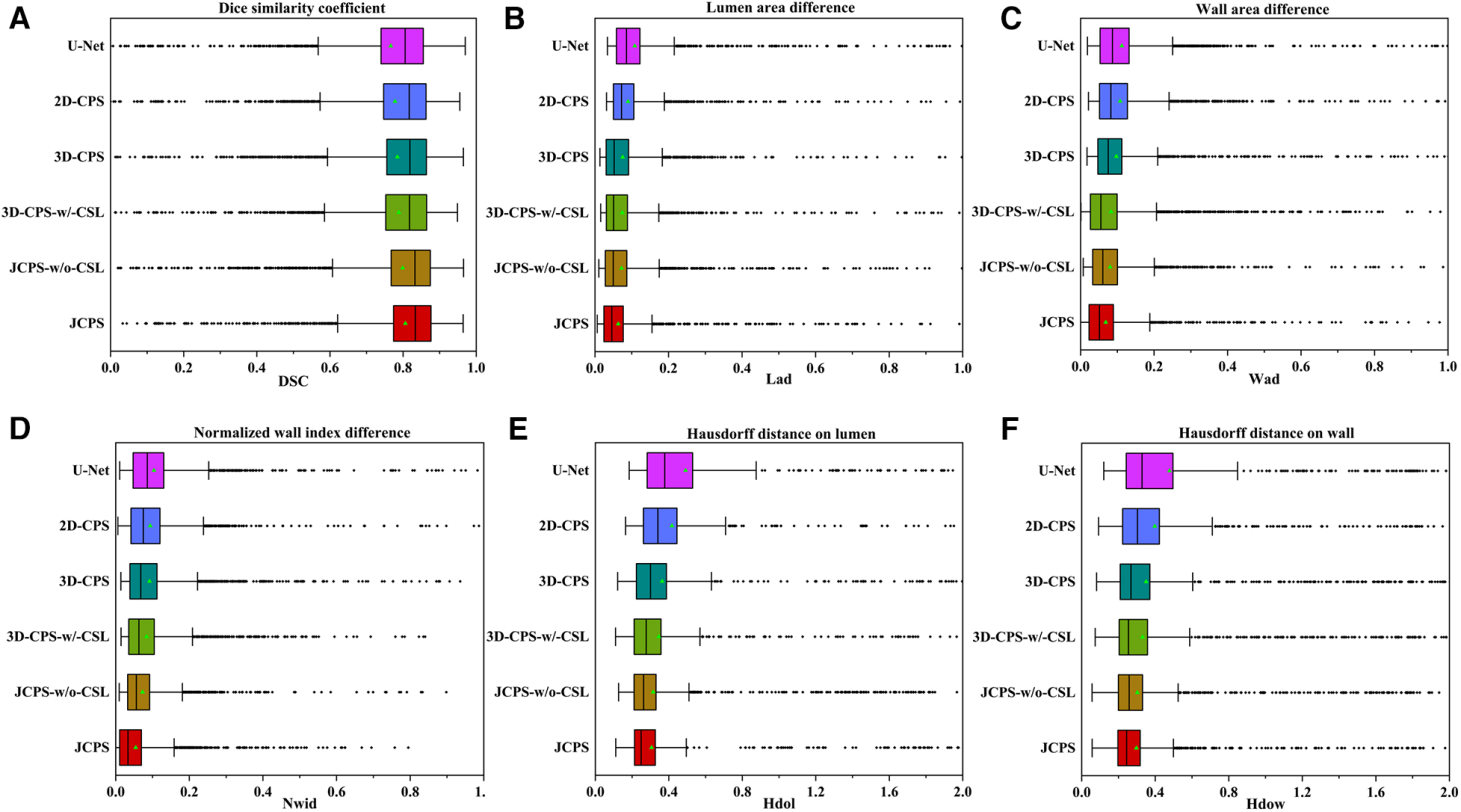
Performance metrics (DSC, Lad, Wad, Nwid, Hdol, Hdow) for JCPS and its variants, where ♦ indicates outliers. Note that the proportion of outliers in each evaluation index of our method is basically less than 3%.

**Figure 9:**
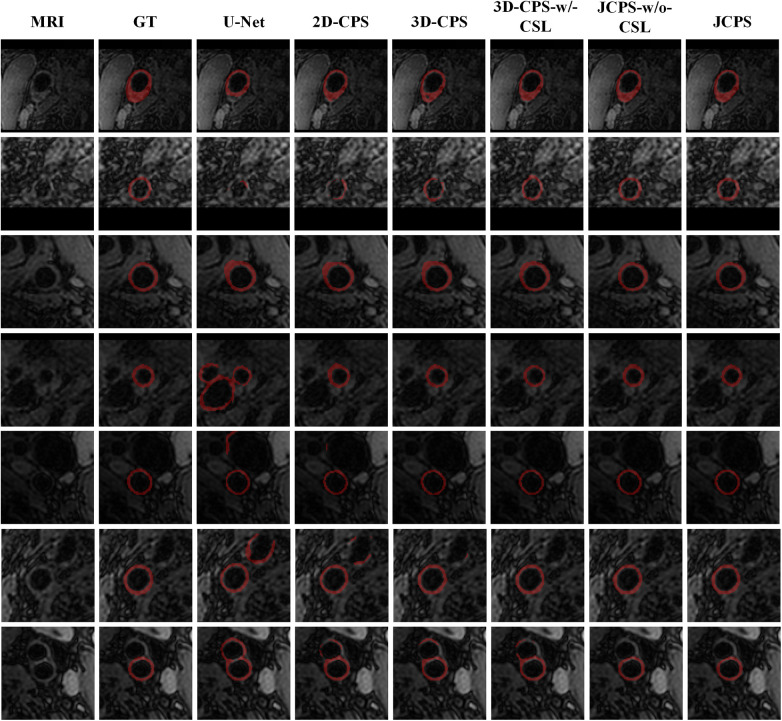
Visual comparison between JCPS and its variants, where the second column GT represents the ground truth, referring to the high-quality annotations. These red annotations represent the segmentation of the vessel wall. The case of blood vessels with the plaque are shown on the first row, blood vessels with fuzzy boundary issues are shown in the second through fifth rows, and images of the carotid artery bifurcation are shown in the sixth and seventh rows.

The effectiveness of each component in our method is demonstrated in Table 2 and Figure 8. First, we examined the effectiveness of the semi-supervised method CPS by comparing U-Net and 2D–CPS during the fine segmentation stage. The results presented in Table 2 and Figure 8 indicate a significant improvement in segmentation accuracy with 2D-CPS compared to U-Net, as evidenced by higher scores across all indicators. This suggests that the CPS network is better suited for datasets with limited labeled data and exhibits superior generalization performance. In addition, the visualization results depicted in Figure 9 demonstrate a substantial enhancement in our segmentation accuracy for images containing lesions and those near the carotid bifurcation, surpassing the performance of plain U-Net models. This further confirmed the effectiveness of CPS in improving segmentation accuracy.

**Table 2:** Performance comparison of the carotid vessel wall segmentation between JCPS and its variants.

Method	DSC	${\mathrm{DSC}}^{L}$	${\mathrm{DSC}}^{W}$	Lad	Wad	Nwid	Hdol	Hdow	QS
U-Net	0.766	0.908	0.911	0.108	0.112	0.104	0.491	0.478	0.791
2D-CPS	0.778	0.924	0.927	0.090	0.108	0.094	0.416	0.397	0.810
3D-CPS	0.784	0.938	0.933	0.075	0.097	0.092	0.364	0.350	0.821
3D-CPS-w/-CSL	0.788	0.938	0.934	0.074	0.083	0.084	0.346	0.331	0.828
JCPS-w/o-CSL	0.799	0.937	0.935	0.072	0.080	0.073	0.315	0.303	0.839
JCPS	**0.806**	**0.939**	**0.939**	**0.063**	**0.068**	**0.054**	**0.305**	**0.297**	**0.850**

U-Net: consists of a vascular center-of-gravity positioning model and a 2D U-Net model. 2D-CPS: consists of a vascular center-of-gravity positioning model and a 2D CPS model. 3D-CPS: consists of a vascular center-of-gravity positioning model and a 3D CPS model. 3D-CPS-w/-CSL: consists of a vascular center-of-gravity positioning model and a 3D CPS model with continuous supervision loss. JCPS-w/o-CSL: consists of a vascular center-of-gravity positioning model and a joint 2D–3D CPS model without continuous supervision loss.

The bold values represent the optimal results achieved in the respective columns for the indicators.

Through the comparison between 3D-CPS and 2D-CPS, it can be concluded that the 3D CPS network yields superior results by leveraging the information across slices. As shown in Table 2, Figures 8 and 9, the 3D-CPS outperforms the 2D-CPS in the fine segmentation stage, which produced more complete contours for the challenging images, showing the improvement brought by the 3D segmentation approaches.

We then investigated the performance of the joint 2D–3D CPS model. By using the same loss function as the 3D-CPS model, the JCPS-w/o-CSL demonstrated a significant enhancement in segmentation accuracy and yielded superior results for challenging images (refer to Table 2, Figures 8 and 9). It verified the effectiveness of using 2D CPS to generate high-quality pseudo labels that aid the 3D CPS networks in achieving accurate segmentation. Furthermore, it showcases that the joint 2D–3D semi-supervised network is well-suited for processing 3D carotid image datasets with limited 2D labels available.

**Figure 10:**
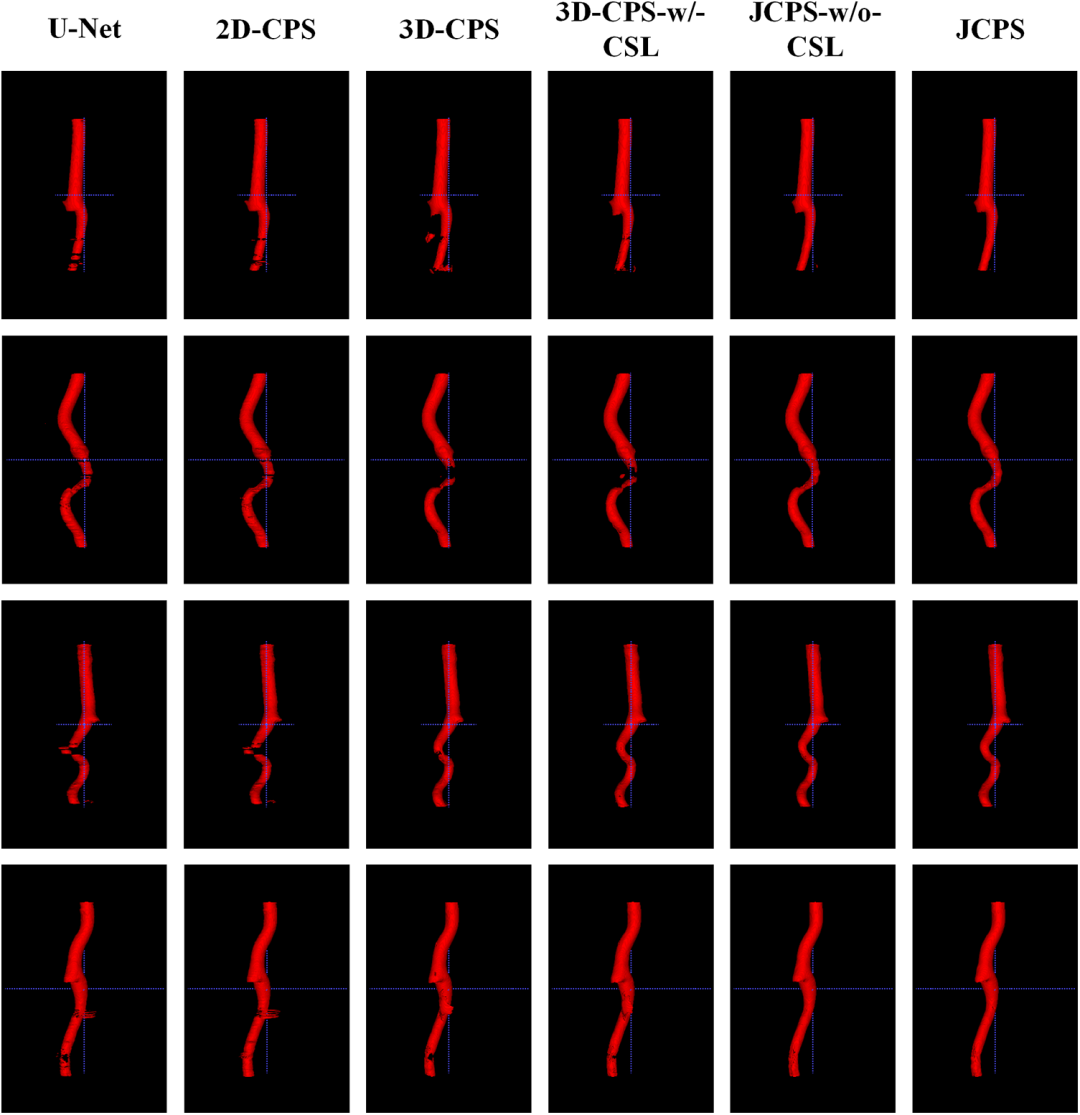
3D visualization comparison between the JCPS and its variants, where red annotations represent the segmentation of the vessel wall.

Finally, we introduced the continuous supervision loss into the joint 2D–3D CPS network to ensure the continuity between adjacent slices. Through a comparison between 3D-CPS, 3D-CPS-w/-CSL, JCPS-w/o-CSL, and JCPS, it was observed that 3D-CPS-w/-CSL exhibited slightly better performance across all metrics (refer to Table 2 and Figure 8). In addition, Figure 9 illustrated that 3D-CPS-w/-CSL achieved superior segmentation results compared to 3D-CPS, but both were slightly inferior to JCPS-w/o-CSL. Furthermore, the visualization of 3D carotid vessel wall segmentation in Figure 10 demonstrated that JCPS outperformed JCPS-w/o-CSL in certain details, indicating the beneficial effect of CSL in obtaining a more continuous carotid vessel wall segmentation.

Based on the visualization results depicted in Figure 9, it was observed that all methods were able to accurately identify the vessel region of interest by utilizing the vascular center of gravity obtained during the initial coarse segmentation stage. This indicates the feasibility of the vascular center-of-gravity positioning model. The first two rows of Figure 9 demonstrate that the segmentation methods encountered challenges with under-segmentation when dealing with vessel images featuring blurred boundaries and plaques. However, our method successfully mitigated these issues by leveraging the semi-supervised learning approach and ensuring continuity between adjacent layers. Consequently, our method achieved stable and precise segmentation outcomes. Furthermore, in the third and fourth rows, it was also noted that images of blood vessels with indistinct boundaries could lead to over-segmentation. Nevertheless, our approach effectively addressed such cases. In the last three examples, it was evident that accurately segmenting the target artery near the carotid bifurcation posed difficulties for other methods due to limited labeled data. Remarkably, our method overcame this problem in most instances.

The 3D visualization results of our method and its variants are shown in Figure 10. Compared to methods using 3D networks, both U-Net and 2D-CPS produced discontinuous and incomplete blood vessels. By looking at the middle two columns, we saw that 3D-CPS provided a more complete vascular structure but might still fail in some challenging regions for such a problem with the dataset of incomplete labels. Also, it can be clearly observed that our JCPS could estimate complete and reasonable 3D segmentation results with fewer areas of poor segmentation quality.

### 3.4. Graphical user interface

**Figure 11:**
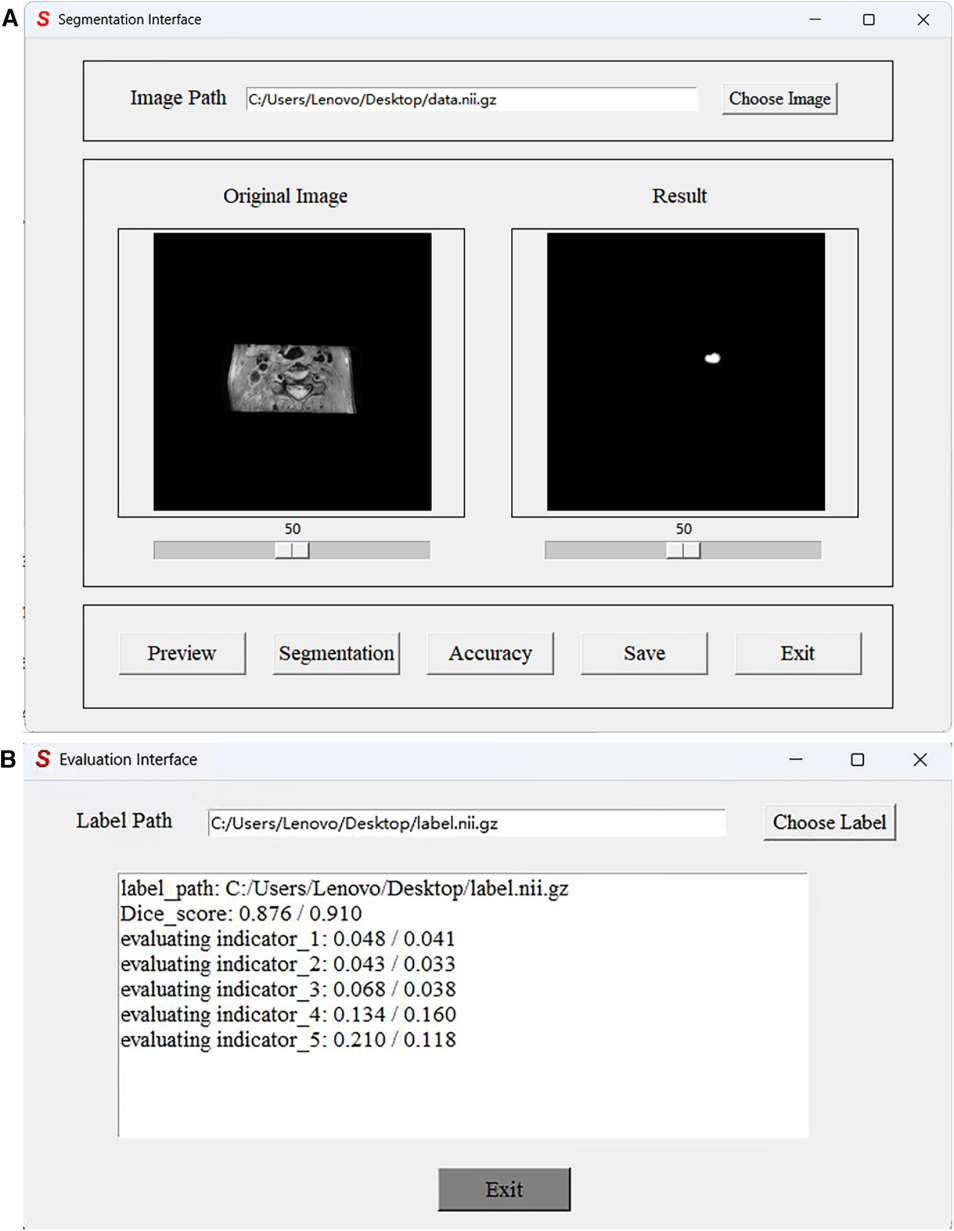
Graphical User Interface of the JCPS method. (**A**) The vessel segmentation interface can display the original image, run the JCPS method, and display and save the segmentation results. (**B**) The evaluation interface can provide six indicators to illustrate the segmentation accuracy of rough segmentation and fine segmentation.

In practical applications, end-users exhibit a preference toward software solutions that are user-friendly and incorporate a GUI. However, to the best of our knowledge, a comprehensive human–computer interaction (HCI) system that is exclusively dedicated to MRI black-blood carotid image segmentation and offers an effective HCI verification environment for current deep learning algorithms has yet to be established, thereby significantly limiting the clinical application of these algorithms. To address this limitation, we have developed a complete automatic vessel segmentation system founded on a deep learning model. Our system encompasses essential functions including data reading, model import, vessel segmentation, result display, and segmentation accuracy evaluation, seamlessly integrated in a pipeline fashion. The system was implemented using the Python programming language, and the vessel segmentation model based on deep learning algorithms could be executed on a single machine. Figure 11 demonstrates the GUI interface used for both coarse and fine segmentation of the lumen and outer wall. It allows for the visualization of segmentation results and evaluation indicators, thereby illustrating the accuracy of the segmentation process. We tested the CPU time to process one black-blood MRI data of size 230×720×720 on an AMD Ryzen 7 5700U processor. The CPU time for coarse segmentation and fine segmentation is 80.73 and 139.42 s, respectively, which can satisfy the clinical needs.

## 4. Concluding remarks

In this study, we developed a two-stage segmentation framework for carotid vessel wall segmentation. In the coarse segmentation stage, we achieved automatic detection of the vascular center of gravity using a vascular center-of-gravity positioning model. The original images were then clipped into local patches containing vessels based on centers of gravity and used as inputs for fine segmentation modeling. Notably, our proposed approach enabled accurate localization of vascular center-of-gravity without any manual intervention. In the fine segmentation stage, we employed the joint 2D–3D CPS network to estimate the vessel wall. To ensure accurate segmentation of vascular structures, we introduced a novel hybrid loss function. In comparison to the existing approaches, our method did not require a large amount of labeled data and human interaction, and it exhibited improved segmentation performance across a range of evaluation indicators. Therefore, with reduced costs, the proposed JCPS network could facilitate clinicians in reading vessel wall outlines and diagnosing atherosclerosis. Moreover, a user-friendly and effective graphical user interface has been created to simplify the implementation of our carotid vessel wall segmentation method.

Our JCPS can handle the task of segmenting the carotid artery vessel wall with low image qualities. Indeed, our fine segmentation network has quite good robustness to the results of coarse segmentation, which can provide reasonable segmentation results even for coarse segmentation with defects. However, its performance may deteriorate when dealing with other vessel segmentation problems. In the future, we plan to explore the domain adaptive coarse segmentation model to achieve constant performance on different vessel segmentation tasks. On the other hand, the two-stage approach we used has high complexity and the segmentation results also lack interpretability. Thus, we would like to consider incorporating more effective domain knowledge to develop reliable vessel stenosis prediction methods.

Indeed, our JCPS method is not restricted to carotid black-blood MRI images but also can be used for other blood vessel segmentation and 3D vessel reconstruction tasks. In future works, we would like to investigate automatic segmentation methods depending on even fewer manual annotations for facilitating medical diagnosis. An avenue we plan to pursue involves developing efficient methods for vessel segmentation based on few-shot learning (47) and zero-shot learning (48). In addition, we also intend to evaluate carotid stenosis on the basis of a vascular model combined with hemodynamic simulation.

## Data Availability

The original contributions presented in the study are included in the article/Supplementary material, further inquiries can be directed to the corresponding authors.
